# Supplementary material for: The outcomes of lockdown in the higher education sector during the COVID-19 pandemic
Source: PLoS One. 2023 Apr 25;18(4):e0282907. doi: 10.1371/journal.pone.0282907 (PMC10128953; doi:10.1371/journal.pone.0282907)
Supplement: S1 Data — (PDF) [file pone.0282907.s002.pdf]

Major	Grade	Class	No.	Sex	Teaching Year	Age	Cell Biology	Advanced Math
Clinical medicine	2018	1	1	Male	2019	20	75.00	62.00
Clinical medicine	2018	1	2	Male	2019	21	63.00	78.00
Clinical medicine	2018	1	3	Male	2019	20	#NULL!	#NULL!
Clinical medicine	2018	1	4	Female	2019	20	#NULL!	#NULL!
Clinical medicine	2018	1	5	Male	2019	20	#NULL!	#NULL!
Clinical medicine	2018	1	6	Female	2019	20	#NULL!	#NULL!
Clinical medicine	2018	1	7	Female	2019	20	#NULL!	#NULL!
Clinical medicine	2018	1	8	Female	2019	18	#NULL!	#NULL!
Clinical medicine	2018	1	9	Female	2019	19	84.00	78.00
Clinical medicine	2018	1	10	Female	2019	19	87.00	80.00
Clinical medicine	2018	1	11	Female	2019	20	90.00	73.00
Clinical medicine	2018	1	12	Female	2019	18	90.00	92.00
Clinical medicine	2018	1	13	Female	2019	20	76.00	67.00
Clinical medicine	2018	1	14	Female	2019	19	92.00	74.00
Clinical medicine	2018	1	15	Female	2019	19	70.00	60.00
Clinical medicine	2018	1	16	Female	2019	19	83.00	77.00
Clinical medicine	2018	1	17	Female	2019	20	90.00	70.00
Clinical medicine	2018	1	18	Female	2019	18	85.00	92.00
Clinical medicine	2018	1	19	Female	2019	20	87.00	64.00
Clinical medicine	2018	1	20	Female	2019	19	68.00	84.00
Clinical medicine	2018	1	21	Female	2019	23	89.00	74.00
Clinical medicine	2018	1	22	Female	2019	19	72.00	94.00
Clinical medicine	2018	1	23	Male	2019	18	84.00	75.00
Clinical medicine	2018	1	24	Male	2019	20	81.00	80.00
Clinical medicine	2018	1	25	Male	2019	20	72.00	64.00
Clinical medicine	2018	1	26	Male	2019	20	90.00	80.00
Clinical medicine	2018	1	27	Male	2019	20	75.00	75.00
Clinical medicine	2018	1	28	Male	2019	19	62.00	73.00
Clinical medicine	2018	1	29	Male	2019	20	64.00	85.00
Clinical medicine	2018	1	30	Male	2019	19	73.00	72.00
Clinical medicine	2018	1	31	Male	2019	21	71.00	77.00
Clinical medicine	2018	1	32	Male	2019	21	93.00	77.00
Clinical medicine	2018	1	33	Male	2019	19	90.00	84.00
Clinical medicine	2018	1	34	Male	2019	19	83.00	85.00
Clinical medicine	2018	1	35	Male	2019	20	63.00	79.00
Clinical medicine	2018	1	36	Male	2019	19	70.00	60.00
Clinical medicine	2018	1	37	Male	2019	20	85.00	80.00
Clinical medicine	2018	1	38	Male	2019	20	78.00	73.00
Clinical medicine	2018	1	39	Male	2019	19	75.00	67.00
Clinical medicine	2018	1	40	Male	2019	20	77.00	84.00
Clinical medicine	2018	1	41	Male	2019	19	67.00	57.00
Clinical medicine	2018	2	1	Male	2019	21	75.00	80.00
Clinical medicine	2018	2	2	Female	2019	20	#NULL!	#NULL!
Clinical medicine	2018	2	3	Female	2019	20	#NULL!	#NULL!
Clinical medicine	2018	2	4	Female	2019	21	#NULL!	#NULL!

Clinical medicine	2018	2	5	Male	2019	19	#NULL!	#NULL!
Clinical medicine	2018	2	6	Female	2019	19	#NULL!	#NULL!
Clinical medicine	2018	2	7	Female	2019	20	#NULL!	#NULL!
Clinical medicine	2018	2	8	Female	2019	20	75.00	57.00
Clinical medicine	2018	2	9	Female	2019	19	94.00	84.00
Clinical medicine	2018	2	10	Female	2019	20	85.00	84.00
Clinical medicine	2018	2	11	Female	2019	18	76.00	68.00
Clinical medicine	2018	2	12	Female	2019	19	86.00	86.00
Clinical medicine	2018	2	13	Female	2019	19	84.00	73.00
Clinical medicine	2018	2	14	Female	2019	19	83.00	73.00
Clinical medicine	2018	2	15	Female	2019	20	69.00	62.00
Clinical medicine	2018	2	16	Female	2019	19	78.00	85.00
Clinical medicine	2018	2	17	Female	2019	19	78.00	70.00
Clinical medicine	2018	2	18	Female	2019	19	93.00	92.00
Clinical medicine	2018	2	19	Female	2019	19	77.00	78.00
Clinical medicine	2018	2	20	Female	2019	19	78.00	70.00
Clinical medicine	2018	2	21	Female	2019	19	78.00	69.00
Clinical medicine	2018	2	22	Female	2019	19	83.00	93.00
Clinical medicine	2018	2	23	Female	2019	19	80.00	90.00
Clinical medicine	2018	2	24	Male	2019	19	78.00	78.00
Clinical medicine	2018	2	25	Male	2019	21	90.00	95.00
Clinical medicine	2018	2	26	Male	2019	20	87.00	96.00
Clinical medicine	2018	2	27	Male	2019	20	77.00	73.00
Clinical medicine	2018	2	28	Male	2019	19	69.00	60.00
Clinical medicine	2018	2	29	Male	2019	20	74.00	64.00
Clinical medicine	2018	2	30	Male	2019	21	77.00	69.00
Clinical medicine	2018	2	31	Male	2019	19	75.00	68.00
Clinical medicine	2018	2	32	Male	2019	21	71.00	65.00
Clinical medicine	2018	2	33	Male	2019	20	77.00	69.00
Clinical medicine	2018	2	34	Male	2019	20	78.00	74.00
Clinical medicine	2018	2	35	Male	2019	20	95.00	95.00
Clinical medicine	2018	2	36	Male	2019	19	83.00	90.00
Clinical medicine	2018	2	37	Male	2019	19	70.00	77.00
Clinical medicine	2018	2	38	Male	2019	19	86.00	79.00
Clinical medicine	2018	2	39	Male	2019	20	65.00	71.00
Clinical medicine	2018	2	40	Male	2019	20	41.00	34.00
Clinical medicine	2019	1	1	Female	2020	18	#NULL!	#NULL!
Clinical medicine	2019	1	2	Male	2020	21	#NULL!	#NULL!
Clinical medicine	2019	1	3	Male	2020	19	#NULL!	#NULL!
Clinical medicine	2019	1	4	Female	2020	19	#NULL!	#NULL!
Clinical medicine	2019	1	5	Male	2020	19	#NULL!	#NULL!
Clinical medicine	2019	1	6	Male	2020	19	#NULL!	#NULL!
Clinical medicine	2019	1	7	Male	2020	19	#NULL!	#NULL!
Clinical medicine	2019	1	8	Female	2020	20	#NULL!	#NULL!
Clinical medicine	2019	1	9	Female	2020	19	#NULL!	#NULL!
Clinical medicine	2019	1	10	Female	2020	19	#NULL!	#NULL!
Clinical medicine	2019	1	11	Male	2020	19	#NULL!	#NULL!
Clinical medicine	2019	1	12	Male	2020	20	#NULL!	#NULL!

Clinical medicine	2019	1	13	Male	2020	19	#NULL!	#NULL!
Clinical medicine	2019	1	14	Female	2020	20	#NULL!	#NULL!
Clinical medicine	2019	1	15	Female	2020	20	#NULL!	#NULL!
Clinical medicine	2019	1	16	Female	2020	21	#NULL!	#NULL!
Clinical medicine	2019	1	17	Female	2020	19	#NULL!	#NULL!
Clinical medicine	2019	1	18	Female	2020	20	73.00	71.00
Clinical medicine	2019	1	19	Female	2020	19	70.00	79.00
Clinical medicine	2019	1	20	Male	2020	19	65.00	76.00
Clinical medicine	2019	1	21	Male	2020	24	74.00	79.00
Clinical medicine	2019	1	22	Female	2020	18	78.00	73.00
Clinical medicine	2019	1	23	Male	2020	19	74.00	81.00
Clinical medicine	2019	1	24	Female	2020	19	86.00	89.00
Clinical medicine	2019	1	25	Female	2020	19	75.00	74.00
Clinical medicine	2019	1	26	Female	2020	19	88.00	72.00
Clinical medicine	2019	1	27	Male	2020	19	58.00	71.00
Clinical medicine	2019	1	28	Male	2020	21	91.00	99.00
Clinical medicine	2019	1	29	Male	2020	19	89.00	93.00
Clinical medicine	2019	1	30	Female	2020	19	89.00	81.00
Clinical medicine	2019	1	31	Female	2020	19	73.00	73.00
Clinical medicine	2019	1	32	Female	2020	20	86.00	93.00
Clinical medicine	2019	1	33	Male	2020	19	88.00	88.00
Clinical medicine	2019	1	34	Male	2020	19	69.00	62.00
Clinical medicine	2019	1	35	Male	2020	20	83.00	80.00
Clinical medicine	2019	1	36	Male	2020	19	79.00	81.00
Clinical medicine	2019	1	37	Female	2020	20	85.00	77.00
Clinical medicine	2019	1	38	Female	2020	19	80.00	78.00
Clinical medicine	2019	1	39	Male	2020	19	65.00	72.00
Clinical medicine	2019	1	40	Male	2020	20	83.00	87.00
Clinical medicine	2019	1	41	Female	2020	20	89.00	89.00
Clinical medicine	2019	1	42	Male	2020	20	81.00	85.00
Clinical medicine	2019	1	43	Female	2020	19	88.00	99.00
Clinical medicine	2019	1	44	Male	2020	19	77.00	73.00
Clinical medicine	2019	1	45	Male	2020	20	79.00	89.00
Clinical medicine	2019	1	46	Male	2020	20	69.00	72.00
Clinical medicine	2019	2	1	Female	2020	19	#NULL!	#NULL!
Clinical medicine	2019	2	2	Male	2020	20	#NULL!	#NULL!
Clinical medicine	2019	2	3	Female	2020	19	#NULL!	#NULL!
Clinical medicine	2019	2	4	Female	2020	18	#NULL!	#NULL!
Clinical medicine	2019	2	5	Female	2020	20	#NULL!	#NULL!
Clinical medicine	2019	2	6	Female	2020	19	#NULL!	#NULL!
Clinical medicine	2019	2	7	Female	2020	19	#NULL!	#NULL!
Clinical medicine	2019	2	8	Female	2020	18	#NULL!	#NULL!
Clinical medicine	2019	2	9	Female	2020	20	#NULL!	#NULL!
Clinical medicine	2019	2	10	Female	2020	19	#NULL!	#NULL!
Clinical medicine	2019	2	11	Female	2020	20	#NULL!	#NULL!
Clinical medicine	2019	2	12	Female	2020	20	#NULL!	#NULL!
Clinical medicine	2019	2	13	Male	2020	20	#NULL!	#NULL!
Clinical medicine	2019	2	14	Female	2020	19	#NULL!	#NULL!

Clinical medicine	2019	2	15	Male	2020	20	#NULL!	#NULL!
Clinical medicine	2019	2	16	Male	2020	19	65.00	73.00
Clinical medicine	2019	2	17	Female	2020	20	85.00	79.00
Clinical medicine	2019	2	18	Male	2020	21	71.00	81.00
Clinical medicine	2019	2	19	Male	2020	20	69.00	84.00
Clinical medicine	2019	2	20	Male	2020	20	74.00	87.00
Clinical medicine	2019	2	21	Female	2020	21	77.00	77.00
Clinical medicine	2019	2	22	Female	2020	20	88.00	85.00
Clinical medicine	2019	2	23	Male	2020	20	55.00	78.00
Clinical medicine	2019	2	24	Female	2020	18	78.00	88.00
Clinical medicine	2019	2	25	Male	2020	21	62.00	74.00
Clinical medicine	2019	2	26	Male	2020	19	80.00	86.00
Clinical medicine	2019	2	27	Male	2020	20	61.00	41.00
Clinical medicine	2019	2	28	Male	2020	19	83.00	87.00
Clinical medicine	2019	2	29	Female	2020	19	76.00	89.00
Clinical medicine	2019	2	30	Male	2020	20	54.00	60.00
Clinical medicine	2019	2	31	Male	2020	19	81.00	81.00
Clinical medicine	2019	2	32	Male	2020	19	60.00	64.00
Clinical medicine	2019	2	33	Female	2020	20	88.00	78.00
Clinical medicine	2019	2	34	Male	2020	20	73.00	62.00
Clinical medicine	2019	2	35	Female	2020	19	75.00	67.00
Clinical medicine	2019	2	36	Female	2020	19	90.00	89.00
Clinical medicine	2019	2	37	Female	2020	19	74.00	74.00
Clinical medicine	2019	2	38	Male	2020	19	80.00	83.00
Clinical medicine	2019	2	39	Female	2020	19	70.00	84.00
Clinical medicine	2019	2	40	Female	2020	22	85.00	83.00
Clinical medicine	2019	2	41	Female	2020	19	88.00	81.00
Clinical medicine	2019	2	42	Female	2020	19	91.00	96.00
Clinical medicine	2019	2	43	Female	2020	19	60.00	60.00
Clinical medicine	2019	2	44	Female	2020	19	87.00	80.00
Clinical medicine	2019	2	45	Female	2020	18	82.00	81.00

Chemistry	Physiology	Types	English	Computer	Chinese History	Sports
66.00	67.00	Pre-COVID-19	73.00	79.00	77.00	81.00
77.00	67.00	Pre-COVID-19	64.00	#NULL!	#NULL!	89.00
#NULL!	#NULL!	Pre-COVID-19	#NULL!	87.00	79.00	84.00
#NULL!	#NULL!	Pre-COVID-19	74.00	80.00	85.00	82.00
#NULL!	#NULL!	Pre-COVID-19	65.00	81.00	81.00	72.00
#NULL!	#NULL!	Pre-COVID-19	#NULL!	83.00	80.00	98.00
#NULL!	#NULL!	Pre-COVID-19	75.00	78.00	85.00	80.00
#NULL!	#NULL!	Pre-COVID-19	#NULL!	91.00	89.00	96.00
79.00	79.00	Pre-COVID-19	73.00	88.00	88.00	84.00
89.00	85.00	Pre-COVID-19	78.00	96.00	83.00	88.00
90.00	89.00	Pre-COVID-19	71.00	91.00	79.00	86.00
81.00	89.00	Pre-COVID-19	70.00	85.00	85.00	88.00
70.00	82.00	Pre-COVID-19	78.00	73.00	77.00	83.00
84.00	81.00	Pre-COVID-19	74.00	94.00	84.00	86.00
67.00	68.00	Pre-COVID-19	#NULL!	83.00	76.00	76.00
67.00	74.00	Pre-COVID-19	77.00	83.00	86.00	88.00
84.00	79.00	Pre-COVID-19	81.00	93.00	80.00	78.00
87.00	85.00	Pre-COVID-19	80.00	93.00	82.00	83.00
83.00	85.00	Pre-COVID-19	#NULL!	85.00	79.00	82.00
73.00	64.00	Pre-COVID-19	71.00	91.00	80.00	82.00
84.00	75.00	Pre-COVID-19	72.00	88.00	84.00	80.00
73.00	64.00	Pre-COVID-19	77.00	77.00	77.00	88.00
81.00	76.00	Pre-COVID-19	68.00	86.00	75.00	81.00
82.00	76.00	Pre-COVID-19	73.00	73.00	89.00	81.00
78.00	68.00	Pre-COVID-19	63.00	79.00	78.00	87.00
82.00	94.00	Pre-COVID-19	69.00	80.00	83.00	80.00
81.00	86.00	Pre-COVID-19	64.00	73.00	72.00	87.00
73.00	72.00	Pre-COVID-19	#NULL!	83.00	80.00	90.00
75.00	63.00	Pre-COVID-19	62.00	94.00	80.00	98.00
73.00	68.00	Pre-COVID-19	73.00	87.00	79.00	80.00
74.00	75.00	Pre-COVID-19	63.00	94.00	84.00	87.00
79.00	81.00	Pre-COVID-19	60.00	83.00	86.00	85.00
84.00	85.00	Pre-COVID-19	69.00	79.00	81.00	85.00
78.00	75.00	Pre-COVID-19	76.00	93.00	79.00	88.00
76.00	51.00	Pre-COVID-19	65.00	94.00	71.00	85.00
60.00	60.00	Pre-COVID-19	67.00	75.00	75.00	90.00
71.00	79.00	Pre-COVID-19	64.00	92.00	76.00	90.00
71.00	65.00	Pre-COVID-19	71.00	92.00	79.00	93.00
70.00	63.00	Pre-COVID-19	89.00	74.00	85.00	92.00
85.00	85.00	Pre-COVID-19	73.00	87.00	75.00	88.00
68.00	60.00	Pre-COVID-19	75.00	89.00	74.00	87.00
71.00	82.00	Pre-COVID-19	54.00	88.00	80.00	88.00
#NULL!	#NULL!	Pre-COVID-19	70.00	78.00	90.00	91.00
#NULL!	#NULL!	Pre-COVID-19	76.00	86.00	87.00	91.00
#NULL!	#NULL!	Pre-COVID-19	67.00	81.00	84.00	91.00

#NULL!	#NULL!	Pre-COVID-19	67.00	93.00	79.00	88.00
#NULL!	#NULL!	Pre-COVID-19	67.00	91.00	84.00	81.00
#NULL!	#NULL!	Pre-COVID-19	68.00	73.00	#NULL!	86.00
72.00	63.00	Pre-COVID-19	66.00	71.00	83.00	86.00
81.00	84.00	Pre-COVID-19	75.00	86.00	90.00	84.00
81.00	82.00	Pre-COVID-19	72.00	79.00	83.00	88.00
81.00	70.00	Pre-COVID-19	68.00	74.00	80.00	92.00
86.00	76.00	Pre-COVID-19	77.00	93.00	84.00	84.00
82.00	93.00	Pre-COVID-19	77.00	76.00	83.00	88.00
73.00	83.00	Pre-COVID-19	72.00	79.00	81.00	88.00
64.00	83.00	Pre-COVID-19	79.00	75.00	77.00	80.00
76.00	76.00	Pre-COVID-19	66.00	82.00	82.00	84.00
70.00	60.00	Pre-COVID-19	77.00	85.00	88.00	80.00
78.00	90.00	Pre-COVID-19	71.00	87.00	81.00	84.00
80.00	79.00	Pre-COVID-19	73.00	75.00	81.00	84.00
67.00	68.00	Pre-COVID-19	72.00	81.00	82.00	84.00
71.00	75.00	Pre-COVID-19	#NULL!	91.00	80.00	83.00
81.00	70.00	Pre-COVID-19	74.00	90.00	86.00	84.00
83.00	78.00	Pre-COVID-19	74.00	83.00	82.00	88.00
74.00	80.00	Pre-COVID-19	82.00	82.00	80.00	88.00
82.00	92.00	Pre-COVID-19	66.00	73.00	83.00	85.00
81.00	83.00	Pre-COVID-19	70.00	94.00	86.00	85.00
80.00	89.00	Pre-COVID-19	60.00	85.00	75.00	86.00
70.00	70.00	Pre-COVID-19	60.00	87.00	85.00	90.00
69.00	66.00	Pre-COVID-19	65.00	71.00	73.00	91.00
76.00	49.00	Pre-COVID-19	67.00	88.00	81.00	89.00
71.00	76.00	Pre-COVID-19	65.00	87.00	77.00	85.00
71.00	87.00	Pre-COVID-19	67.00	90.00	79.00	85.00
75.00	81.00	Pre-COVID-19	63.00	88.00	82.00	91.00
76.00	80.00	Pre-COVID-19	65.00	75.00	86.00	85.00
90.00	98.00	Pre-COVID-19	73.00	95.00	82.00	87.00
76.00	76.00	Pre-COVID-19	69.00	73.00	82.00	88.00
71.00	76.00	Pre-COVID-19	66.00	88.00	79.00	87.00
80.00	55.00	Pre-COVID-19	61.00	66.00	78.00	88.00
68.00	51.00	Pre-COVID-19	69.00	73.00	80.00	91.00
65.00	41.00	Pre-COVID-19	60.00	63.00	76.00	85.00
#NULL!	#NULL!	COVID-19	77.00	81.00	85.00	90.00
#NULL!	#NULL!	COVID-19	66.00	83.00	71.00	85.00
#NULL!	#NULL!	COVID-19	#NULL!	86.00	86.00	90.00
#NULL!	#NULL!	COVID-19	86.00	98.00	88.00	82.00
#NULL!	#NULL!	COVID-19	85.00	95.00	83.00	94.00
#NULL!	#NULL!	COVID-19	86.00	97.00	85.00	93.00
#NULL!	#NULL!	COVID-19	83.00	95.00	85.00	86.00
#NULL!	#NULL!	COVID-19	81.00	73.00	85.00	91.00
#NULL!	#NULL!	COVID-19	#NULL!	91.00	86.00	97.00
#NULL!	#NULL!	COVID-19	90.00	94.00	86.00	96.00
#NULL!	#NULL!	COVID-19	86.00	89.00	85.00	95.00
#NULL!	#NULL!	COVID-19	81.00	94.00	86.00	93.00

#NULL!	#NULL!	COVID-19	#NULL!	94.00	86.00	93.00
#NULL!	#NULL!	COVID-19	90.00	92.00	87.00	94.00
#NULL!	#NULL!	COVID-19	81.00	83.00	84.00	76.00
#NULL!	#NULL!	COVID-19	#NULL!	97.00	#NULL!	97.00
#NULL!	#NULL!	COVID-19	87.00	90.00	#NULL!	82.00
83.00	72.00	COVID-19	87.00	96.00	87.00	97.00
81.00	82.00	COVID-19	85.00	90.00	86.00	92.00
61.00	68.00	COVID-19	83.00	87.00	72.00	92.00
72.00	78.00	COVID-19	87.00	91.00	86.00	92.00
78.00	73.00	COVID-19	75.00	87.00	85.00	93.00
74.00	71.00	COVID-19	#NULL!	96.00	86.00	93.00
69.00	81.00	COVID-19	81.00	88.00	86.00	98.00
77.00	80.00	COVID-19	87.00	96.00	87.00	91.00
76.00	72.00	COVID-19	83.00	99.00	88.00	95.00
75.00	69.00	COVID-19	82.00	91.00	86.00	89.00
88.00	82.00	COVID-19	80.00	97.00	86.00	93.00
82.00	74.00	COVID-19	82.00	94.00	85.00	95.00
68.00	83.00	COVID-19	86.00	74.00	84.00	96.00
71.00	78.00	COVID-19	82.00	94.00	88.00	93.00
79.00	79.00	COVID-19	80.00	85.00	86.00	96.00
71.00	76.00	COVID-19	82.00	97.00	87.00	90.00
59.00	63.00	COVID-19	85.00	92.00	86.00	91.00
69.00	73.00	COVID-19	62.00	72.00	85.00	90.00
74.00	76.00	COVID-19	83.00	85.00	83.00	85.00
71.00	70.00	COVID-19	84.00	97.00	87.00	82.00
73.00	80.00	COVID-19	85.00	89.00	88.00	81.00
72.00	68.00	COVID-19	74.00	93.00	87.00	88.00
79.00	75.00	COVID-19	66.00	70.00	86.00	81.00
76.00	73.00	COVID-19	75.00	93.00	88.00	78.00
69.00	63.00	COVID-19	79.00	81.00	84.00	89.00
72.00	74.00	COVID-19	#NULL!	77.00	86.00	92.00
70.00	71.00	COVID-19	82.00	92.00	82.00	92.00
73.00	60.00	COVID-19	85.00	86.00	84.00	93.00
76.00	76.00	COVID-19	84.00	79.00	85.00	90.00
#NULL!	#NULL!	COVID-19	88.00	88.00	86.00	96.00
#NULL!	#NULL!	COVID-19	82.00	86.00	87.00	92.00
#NULL!	#NULL!	COVID-19	81.00	93.00	86.00	98.00
#NULL!	#NULL!	COVID-19	83.00	92.00	87.00	91.00
#NULL!	#NULL!	COVID-19	85.00	92.00	87.00	94.00
#NULL!	#NULL!	COVID-19	81.00	83.00	85.00	89.00
#NULL!	#NULL!	COVID-19	85.00	94.00	87.00	94.00
#NULL!	#NULL!	COVID-19	#NULL!	97.00	86.00	92.00
#NULL!	#NULL!	COVID-19	84.00	0.00	83.00	94.00
#NULL!	#NULL!	COVID-19	85.00	89.00	84.00	90.00
#NULL!	#NULL!	COVID-19	78.00	94.00	89.00	88.00
#NULL!	#NULL!	COVID-19	#NULL!	85.00	86.00	75.00
#NULL!	#NULL!	COVID-19	80.00	92.00	87.00	93.00
#NULL!	#NULL!	COVID-19	80.00	89.00	87.00	75.00

#NULL!	#NULL!	COVID-19	#NULL!	93.00	87.00	85.00
70.00	69.00	COVID-19	83.00	86.00	87.00	91.00
78.00	78.00	COVID-19	75.00	93.00	88.00	87.00
76.00	64.00	COVID-19	79.00	89.00	87.00	93.00
71.00	76.00	COVID-19	81.00	84.00	87.00	89.00
70.00	74.00	COVID-19	80.00	89.00	86.00	89.00
76.00	84.00	COVID-19	78.00	83.00	86.00	94.00
68.00	70.00	COVID-19	81.00	78.00	85.00	93.00
65.00	70.00	COVID-19	84.00	83.00	85.00	89.00
69.00	78.00	COVID-19	75.00	94.00	86.00	93.00
73.00	60.00	COVID-19	80.00	95.00	84.00	90.00
72.00	72.00	COVID-19	68.00	79.00	83.00	90.00
64.00	60.00	COVID-19	73.00	90.00	82.00	86.00
77.00	74.00	COVID-19	79.00	87.00	83.00	89.00
72.00	75.00	COVID-19	69.00	99.00	86.00	96.00
71.00	67.00	COVID-19	61.00	24.00	82.00	89.00
78.00	66.00	COVID-19	79.00	91.00	86.00	94.00
68.00	70.00	COVID-19	87.00	99.00	86.00	94.00
66.00	75.00	COVID-19	76.00	88.00	87.00	92.00
73.00	58.00	COVID-19	67.00	73.00	82.00	93.00
59.00	72.00	COVID-19	80.00	95.00	82.00	96.00
74.00	74.00	COVID-19	76.00	95.00	86.00	94.00
65.00	67.00	COVID-19	88.00	94.00	87.00	93.00
77.00	78.00	COVID-19	79.00	90.00	86.00	87.00
66.00	74.00	COVID-19	75.00	78.00	87.00	92.00
83.00	83.00	COVID-19	78.00	87.00	86.00	90.00
78.00	83.00	COVID-19	78.00	86.00	87.00	88.00
81.00	85.00	COVID-19	#NULL!	97.00	86.00	93.00
59.00	61.00	COVID-19	64.00	80.00	85.00	76.00
72.00	77.00	COVID-19	76.00	98.00	86.00	93.00
83.00	71.00	COVID-19	81.00	95.00	87.00	96.00

Biochemistry	Anatomy	Histology	Histo Practice	Histo Practice daily	Histo Practice midterm	Histo Practice final
67.00	77.00	72.00	83.00	98.00	78.00	74.50
47.00	63.00	#NULL!	76.00	79.70	80.00	69.70
73.00	79.00	74.00	81.00	94.90	74.50	74.50
70.00	72.00	72.00	76.00	93.40	52.50	80.60
49.00	69.00	64.00	78.00	93.80	74.00	69.40
67.00	60.00	64.00	83.00	94.30	88.50	71.00
57.00	65.00	69.00	72.00	92.78	60.50	65.30
68.00	66.00	79.00	85.00	95.30	82.50	78.50
73.00	87.00	76.00	86.00	98.80	64.00	93.50
85.00	82.00	87.00	92.00	95.70	98.00	85.80
88.00	87.00	83.00	91.00	97.60	96.00	83.50
78.00	86.00	80.00	91.00	97.30	92.00	85.80
66.00	67.00	67.00	84.00	98.50	85.00	73.10
86.00	82.00	90.00	89.00	97.10	93.50	80.70
67.00	74.00	78.00	86.00	95.50	85.50	78.10
72.00	65.00	70.00	82.00	97.30	77.00	73.20
78.00	81.00	82.00	91.00	95.80	96.00	84.20
85.00	79.00	81.00	91.00	97.10	91.00	85.70
76.00	79.00	75.00	86.00	97.60	82.00	81.10
72.00	69.00	69.00	77.00	96.10	64.00	73.40
76.00	84.00	78.00	87.00	97.00	89.50	76.70
76.00	82.00	82.00	90.00	96.50	91.00	85.50
79.00	62.00	73.00	85.00	98.00	80.50	77.60
71.00	60.00	70.00	79.00	96.20	76.00	67.20
60.00	63.00	53.00	71.00	94.40	52.50	66.70
84.00	79.00	84.00	87.00	97.00	91.00	76.30
67.00	60.00	64.00	83.00	95.20	90.00	68.20
60.00	60.00	62.00	84.00	98.00	85.00	71.80
64.00	54.00	56.00	81.00	97.50	74.00	74.40
70.00	61.00	70.00	86.00	91.50	89.00	78.60
62.00	61.00	70.00	80.00	97.80	81.00	66.30
76.00	82.00	85.00	92.00	98.00	93.00	87.50
85.00	84.00	84.00	92.00	99.00	92.50	87.40
67.00	65.00	77.00	86.00	96.80	78.50	83.10
63.00	60.00	63.00	66.00	92.80	41.50	65.30
56.00	54.00	54.00	68.00	95.10	37.00	71.50
74.00	73.00	65.00	85.00	94.80	86.00	76.00
71.00	60.00	73.00	77.00	91.30	68.50	71.80
64.00	66.00	74.00	74.00	98.40	66.00	62.30
74.00	70.00	67.00	79.00	94.90	75.00	70.70
60.00	61.00	66.00	78.00	96.60	70.00	69.70
44.00	63.00	44.00	60.00	94.60	24.00	60.60
78.00	74.00	72.00	86.00	97.50	80.00	81.20
78.00	78.00	68.00	81.00	96.60	72.00	75.60
77.00	82.00	74.00	86.00	97.20	82.50	80.40

48.00	63.00	56.00	72.00	94.70	47.50	73.40
83.00	85.00	75.00	85.00	98.80	77.00	79.80
74.00	76.00	77.00	88.00	95.30	81.50	88.10
79.00	70.00	58.00	78.00	95.90	68.50	72.60
85.00	88.00	85.00	97.00	99.50	98.00	93.70
80.00	75.00	71.00	88.00	99.10	78.50	87.80
70.00	68.00	66.00	81.00	98.50	69.00	76.80
87.00	83.00	80.00	91.00	99.40	84.00	90.50
81.00	80.00	83.00	90.00	98.70	85.00	87.90
70.00	66.00	62.00	86.00	99.10	83.50	79.10
61.00	70.00	60.00	74.00	98.10	72.00	58.60
60.00	68.00	62.00	74.00	97.90	61.00	66.90
65.00	79.00	69.00	90.00	98.90	89.00	84.90
89.00	85.00	85.00	95.00	99.30	91.00	95.00
75.00	63.00	71.00	86.00	99.00	69.00	89.80
65.00	70.00	68.50	80.00	98.70	73.00	70.70
75.00	66.00	69.00	84.00	99.00	81.00	76.20
85.00	69.00	66.00	80.00	96.40	61.50	82.20
81.00	79.00	73.00	82.00	98.50	72.00	78.30
77.00	72.00	81.00	84.00	93.30	79.00	81.00
85.00	79.00	76.00	92.00	96.90	87.00	92.70
77.00	80.00	72.00	88.00	96.20	82.00	87.10
56.50	63.00	60.00	69.00	92.60	61.00	58.30
64.00	62.00	51.00	69.00	93.90	52.00	63.60
68.00	60.00	61.00	72.00	91.10	53.00	70.80
68.00	76.00	72.00	72.00	91.80	51.50	73.20
68.00	38.00	52.00	64.00	92.80	41.00	58.90
61.50	73.00	69.00	76.00	97.70	54.00	75.30
74.00	70.00	65.00	74.00	96.60	53.50	73.60
71.00	84.00	68.00	83.00	94.30	83.50	73.60
89.00	89.00	85.00	94.00	97.80	95.50	89.50
73.00	69.00	71.00	78.00	95.70	69.00	71.30
69.00	63.00	57.00	72.00	94.10	60.00	65.30
73.00	76.00	70.00	79.00	96.10	71.00	71.00
66.00	60.00	54.00	63.00	93.00	28.50	65.70
44.00	41.00	38.00	50.00	93.30	13.50	44.30
82.00	76.00	81.00	96.00	100.00	96.00	93.00
70.00	80.00	62.00	67.00	97.00	50.00	56.00
75.00	48.00	72.00	86.00	100.00	82.00	79.00
79.00	72.00	69.00	92.00	100.00	95.00	83.00
75.00	66.00	69.00	86.00	99.00	92.00	72.00
86.00	67.00	78.00	90.00	100.00	88.00	85.00
85.00	74.00	79.00	93.00	99.00	95.00	88.00
62.00	68.00	62.00	87.00	98.00	85.00	80.00
83.00	71.00	70.00	93.00	100.00	94.00	88.00
83.00	80.00	79.00	88.00	100.00	84.00	81.00
82.00	70.00	79.00	94.00	99.00	95.00	90.00
63.00	82.00	68.00	88.00	99.00	88.00	79.00

91.00	81.00	84.00	96.00	99.00	94.00	94.00
89.00	76.00	84.00	93.00	100.00	89.00	91.00
82.00	64.00	68.00	92.00	99.00	96.00	84.00
78.00	67.00	75.00	89.00	99.00	88.00	82.00
82.00	77.00	74.00	90.00	100.00	95.00	79.00
77.00	78.00	70.00	92.00	100.00	84.00	92.00
83.00	74.00	74.00	88.00	98.00	87.00	81.00
57.00	70.00	69.00	83.00	98.00	72.00	81.00
82.00	77.00	74.00	86.00	98.00	73.00	87.00
74.00	77.00	75.00	85.00	99.00	77.00	80.00
80.00	76.00	74.00	90.00	99.00	85.00	88.00
83.00	82.00	79.00	93.00	100.00	95.00	87.00
82.00	82.00	79.00	84.00	98.00	90.00	70.00
77.00	69.00	77.00	90.00	100.00	89.00	82.00
64.00	81.00	71.00	71.00	99.00	69.00	52.00
87.00	60.00	76.00	97.00	100.00	97.00	95.00
85.00	80.00	81.00	91.00	100.00	89.00	86.00
76.00	65.00	76.00	91.00	98.00	81.00	92.00
77.00	75.00	71.00	83.00	98.00	69.00	81.00
88.00	77.00	81.00	94.00	99.00	96.00	88.00
84.00	82.00	74.00	89.00	98.00	91.00	81.00
75.00	74.00	72.00	76.00	98.00	66.00	67.00
76.00	60.00	76.00	88.00	99.00	90.00	79.00
76.00	80.00	76.00	91.00	97.00	97.00	83.00
77.00	74.00	77.00	93.00	100.00	84.00	94.00
84.00	76.00	70.00	75.00	97.00	71.00	61.00
64.00	82.00	72.00	79.00	100.00	79.00	63.00
73.00	82.00	71.00	90.00	98.00	92.00	83.00
87.00	80.00	80.00	95.00	99.00	94.00	93.00
68.00	60.00	69.00	84.00	99.00	85.00	71.00
83.00	73.00	79.00	93.00	100.00	95.00	86.00
78.00	68.00	74.00	84.00	98.00	85.00	72.00
80.00	69.00	78.00	85.00	98.00	85.00	76.00
78.00	72.00	70.00	67.00	98.00	45.00	60.00
78.00	81.00	63.00	86.00	99.00	76.00	83.00
89.00	78.00	76.00	91.00	99.00	86.00	89.00
79.00	75.00	78.00	94.00	99.00	91.00	93.00
89.00	82.00	79.00	95.00	98.00	100.00	88.00
86.00	74.00	85.00	97.00	99.00	99.00	93.00
79.00	74.00	65.00	92.00	99.00	90.00	87.00
89.00	77.00	82.00	94.00	99.00	92.00	91.00
72.00	64.00	66.00	86.00	99.00	88.00	74.00
77.00	65.00	81.00	92.00	100.00	94.00	85.00
82.00	79.00	71.00	91.00	99.00	91.00	86.00
84.00	72.00	68.00	93.00	99.00	93.00	89.00
66.00	75.00	55.00	74.00	95.00	61.00	68.00
83.00	80.00	79.00	87.00	99.00	92.00	75.00
81.00	70.00	64.00	92.00	99.00	90.00	87.00

59.00	44.00	60.00	80.00	97.00	69.00	75.00
60.00	74.00	60.00	70.00	94.00	51.00	67.00
79.00	62.00	60.00	89.00	100.00	79.00	87.00
73.00	69.00	62.00	89.00	98.00	79.00	89.00
82.00	73.00	61.00	80.00	98.00	73.00	72.00
74.00	68.00	62.50	77.00	96.00	73.00	65.00
68.00	75.00	65.00	93.00	100.00	89.00	91.00
79.00	76.00	67.00	89.00	99.00	76.00	91.00
68.00	83.00	60.00	75.00	94.00	64.00	69.00
88.00	87.00	73.00	88.00	99.00	73.00	91.00
76.00	87.00	63.50	77.00	98.00	75.00	62.00
81.00	79.00	57.00	80.00	98.00	79.00	67.00
0.00	70.00	0.00	49.00	96.00	25.00	31.00
85.00	67.00	71.00	92.00	99.00	94.00	85.00
82.00	71.00	73.00	85.00	100.00	76.00	80.00
51.00	33.00	48.00	33.00	69.00	9.00	24.00
68.00	65.00	64.00	84.00	99.00	81.00	76.00
62.00	85.00	64.50	63.00	98.00	51.00	46.00
84.00	76.00	74.00	93.00	99.00	95.00	88.00
69.00	76.00	66.00	89.00	98.00	85.00	85.00
72.00	84.00	66.00	91.00	99.00	94.00	83.00
88.00	83.00	78.00	98.00	100.00	97.00	98.00
81.00	80.00	75.00	85.00	99.00	90.00	71.00
71.00	90.00	69.50	72.00	97.00	43.00	76.00
66.00	63.00	62.00	75.00	99.00	67.00	64.00
83.00	82.00	71.00	97.00	100.00	95.00	96.00
85.00	81.00	73.00	91.00	100.00	80.00	93.00
82.00	82.00	67.00	94.00	99.00	91.00	93.00
61.00	72.00	61.00	88.00	99.00	86.00	81.00
89.00	84.00	81.00	97.00	100.00	95.00	95.00
79.00	80.00	61.50	86.00	99.00	77.00	82.00

Histo Practice overall
83.00
76.00
81.00
76.00
78.00
83.00
72.00
85.00
86.00
92.00
91.00
91.00
84.00
89.00
86.00
82.00
91.00
91.00
86.00
77.00
87.00
90.00
85.00
79.00
71.00
87.00
83.00
84.00
81.00
86.00
80.00
92.00
92.00
86.00
66.00
68.00
85.00
77.00
74.00
79.00
78.00
60.00
86.00
81.00
86.00

72.00
85.00
88.00
78.00
97.00
88.00
81.00
91.00
90.00
86.00
74.00
74.00
90.00
95.00
86.00
80.00
84.00
80.00
82.00
84.00
92.00
88.00
69.00
69.00
72.00
72.00
64.00
76.00
74.00
83.00
94.00
78.00
72.00
79.00
63.00
50.00
96.00
67.00
86.00
92.00
86.00
90.00
93.00
87.00
93.00
88.00
94.00
88.00

96.00
93.00
92.00
89.00
90.00
92.00
88.00
83.00
86.00
85.00
90.00
93.00
84.00
90.00
71.00
97.00
91.00
91.00
83.00
94.00
89.00
76.00
88.00
91.00
93.00
75.00
79.00
90.00
95.00
84.00
93.00
84.00
85.00
67.00
86.00
91.00
94.00
95.00
97.00
92.00
94.00
86.00
92.00
91.00
93.00
74.00
87.00
92.00

80.00
70.00
89.00
89.00
80.00
77.00
93.00
89.00
75.00
88.00
77.00
80.00
49.00
92.00
85.00
33.00
84.00
63.00
93.00
89.00
91.00
98.00
85.00
72.00
75.00
97.00
91.00
94.00
88.00
97.00
86.00
