# Supplementary material for: The outcomes of lockdown in the higher education sector during the COVID-19 pandemic
Source: PLoS One. 2023 Apr 25;18(4):e0282907. doi: 10.1371/journal.pone.0282907 (PMC10128953; doi:10.1371/journal.pone.0282907)
Supplement: Attachment — Submitted filename: rebutttal Plos Med3.docx [file pone.0282907.s003.docx]

Dr Xiangjie Kong
Academic Editor
PLOS ONE

PONE-D-22-27104

Dear Dr Kong

We appreciate the constructive comments made by the reviewers, our responses are as follows:

*1. Is the manuscript technically sound, and do the data support the conclusions?
Reviewer #1: Partly*

*Reviewer #2: Yes*

The manuscript is being revised accordingly to improve the quality as specified by reviewer #1 in the following sections.

*2. Has the statistical analysis been performed appropriately and rigorously?*

*Reviewer #1: Yes*

*Reviewer #2: Yes*

Thanks

*3. Have the authors made all data underlying the findings in their manuscript fully available?
Reviewer #1: Yes*

*Reviewer #2: Yes*

Thanks

*4. Is the manuscript presented in an intelligible fashion and written in standard English?
Reviewer #1: Yes*

*Reviewer #2: Yes*

Thanks

5. Review Comments to the Author

*Reviewer #1: This paper reveals to us a very interesting conclusion.*

Thanks

There some suggestions and concerns are listed as following:
*1. a. Is it reasonable to use final grades to measure students' learning outputs?*

This point requires clarification, so we have added the following section in Materials and Methods, it now reads:

“In all subjects, the overall assessment resulting in the final grades consisted of three elements: the Daily Performance, Mid‑term Tests and the Final Test, which followed the criteria for teaching management at Chengdu University. Thus, the final grades reflected the overall performance of the students throughout each term. In addition, as an example, for the subject Histology Practice we have provided detailed data on these three specific elements by applying The Electronic Teaching Management System (Fig. 4), to acquire more detailed academic performance data and carry out a more adequate statistical analysis.” (Materials and Methods).

*b. Is it due to certain differences in the content and difficulty of the exam?*

(2) Agree, we have added the following section in the Discussion, it now reads:

“Notably, it is possible that the differences in performance that we observed may be due to certain difference in the content that was provided to students and/or differences in the difficulty of the assessments that were undertaken. With the possible exception of Sports, the teaching staff involved were confident that both content and assessments were very close to identical between the two cohorts. However, our future studies will verify this point.”

*2. Two specific examples are suggested to illustrate the plausibility of the results of this study, such as comparing learning outcomes by comparing the scores of two groups of students on computer programming questions with similar academic requirements.*

We agree completely. However, the current study was performed 2 years ago in response to a specific emergency situation (the COVID-19 pandemic lockdowns) and the teaching model that has been subsequently changed into a combined online and face-to-face teaching format, partly in response to the data presented in this manuscript. However, we will perform such testing in a future study.

*3. It is recommended that the authors collect and study data of students' self-assessments about their mastery degree of some specific knowledge.*

We appreciate such constructive comments, however, we are not able to pursue this question further, because the students have reached clinical years now, and their depth of clinical perspective in relation to the basics sciences has changed since 2020. Thus, the students’ self-assessments may not be objective or clear to enable the test of this hypothesis. We have added this limitation into the Discussion, it now reads: “Additionally, a useful insight may be gained in future by surveying data concerning the students' self-assessments perception about the extent of their mastery of some specific essential knowledge in each subject, which will be performed in a future study.”

*Reviewer #2: An interesting study that covered majority of the first year medical course subjects undertaken prior to and during COVID-19. I cannot find any fault to it since all have been explained, analysed and discussed very well.*

Thanks

We have modified our manuscript accordingly.

Yours sincerely

Shisan Bao
